# Prediction method of sugarcane important phenotype data based on multi-model and multi-task

**DOI:** 10.1371/journal.pone.0312444

**Published:** 2024-12-13

**Authors:** Jihong Sun, Chen Sun, Zhaowen Li, Ye Qian, Tong Li

**Affiliations:** 1 College of Agronomy and Biotechnology, Yunnan Agricultural University, Kunming, Yunnan, China; 2 The Key Laboratory for Crop Production and Smart Agriculture of Yunnan Province, Yunnan Agricultural University, Kunming, Yunnan, China; 3 Big Data School, Yunnan Agricultural University, Kunming, Yunnan, China; Federal University of Mato Grosso do Sul, BRAZIL

## Abstract

The efficacy of generalized sugarcane yield prediction models holds significant implications for global food security. Given that machine learning algorithms often surpass the precision of remote sensing technology, further exploration of machine learning algorithms in the development of sugarcane yield prediction models is imperative. In this study, we employed six key phenotypic traits of sugarcane, specifically plant height, stem diameter, third-node length (internode length), leaf length, leaf width, and field brix, along with eight machine learning methods: logistic regression, linear regression, K-Nearest Neighbors (KNN), Support Vector Machine (SVM), Backpropagation Neural Network (BPNN), Decision Tree, Random Forest, and the XGBoost algorithm. The aim was to establish an intelligent model ensemble for predicting two crucial phenotypic characteristics—stem diameter and plant height—that determine sugarcane yield, ultimately enhancing the overall yield.The experimental findings indicate that the XGBoost algorithm outperforms the other seven algorithms in predicting these significant phenotypic traits of sugarcane. Furthermore, an analysis of the sugarcane intelligent prediction model’s performance under a specialized data environment, incorporating self-prepared data, reveals that the XGBoost algorithm exhibits greater stability. Notably, the data pertaining to these crucial phenotypic traits have a profound impact on the efficacy of the intelligent models. The research demonstrates that a sugarcane yield prediction model ensemble, incorporating multiple intelligent algorithms, can accurately forecast stem diameter and plant height, thereby predicting sugarcane yield. Additionally, this approach, combined with the principles of sugarcane cross-breeding, provides a valuable reference for the artificial breeding of new sugarcane varieties that excel in stem diameter and plant height, bridging a research gap in indirect yield prediction through sugarcane phenotypic traits.

## Introduction

Sugarcane stands as the preeminent sugar crop globally, serving as a primary source of food and energy for humans. With an annual output exceeding 1.75 billion tons, it ranks among the world’s most extensively cultivated crops. Brazil, the foremost producer in the world, contributes over 700 million tons annually, while India, the second-largest producer, generates more than 300 million tons. China, occupying the fourth position, produces over 200 million tons annually [1]. However, sugarcane’s productivity has been significantly hampered by numerous challenges, including continuous cropping obstacles, diseases and pests, low yield, and lodging. Therefore, accurate prediction of sugarcane yield is paramount for global sugar security.

Crop yield, as a crucial aspect of global food production, constitutes one of the most prevalent research domains among scholars [2]. The development of crop yield prediction models falls into two primary categories: classical empirical models [3–7] and machine learning models [8, 9]. Classical empirical models typically rely on field surveys, biophysical simulations, and statistical frameworks to establish estimation models [10]. In field surveys, seasoned farmers or experts predict crop yields based on field observations. This process demands considerable time and labor, and the effectiveness of these predictions is inherently subjective, relying heavily on the forecaster’s experience. Biophysical models can simulate crop growth stages and yields, providing insights into crop development under diverse meteorological conditions [11, 12]. Nevertheless, these models necessitate rigorous testing and calibration, often requiring a vast array of input parameters, such as soil moisture, meteorological data, and agricultural management information [13]. While the accuracy of such models generally hovers around 70%, they necessitate comprehensive data collection at the scale of the crop-growing area, introducing substantial uncertainty and reducing their predictive performance at larger scales [14]. Statistical models, grounded in probability theory, utilize mathematical statistics to establish functional relationships between variables based on experimental measurements [15]. However, capturing the intricate nonlinear relationships between dependent and independent variables remains a challenge within this framework [16].

In recent scholarly endeavors, numerous studies have demonstrated the superiority of models incorporating multi-intelligent algorithm fusion over traditional field surveys, biophysical models, and statistical models in the context of crop science applications. Within this context, machine learning algorithms can predict crop yields by exploring the nonlinear relationship between influencing factors and crop yields, successfully addressing classification issues and gaining widespread application [17]. The precision of these models is contingent upon the accuracy of the influencing factors and the comprehensiveness of the data. Furthermore, the accuracy of these influencing factors can be retrospectively validated through the construction of verification models utilizing machine learning techniques [18–20]. Currently, machine learning methodologies have gained widespread adoption in forecasting agricultural economic variables [21–24]. Notably, significant achievements have been recorded in the realm of crop yield prediction [25, 26], underscoring the transformative potential of this technology in advancing agricultural predictions and management.

Khaki and Wang [27] formulated a residual neural network model to forecast output, marking a significant advancement in predictive analytics. Mupangwa et al. [28] introduced a long short-term memory (LSTM) model, which seamlessly integrates heterogeneous crop phenology, meteorological, and remote sensing data to predict maize yield at the county level. This innovative model outperforms LASSO and random forest, accounting for 76% of yield variability across the entire corn belt. Khaki et al. [29] furthered this research by developing a convolutional neural network-recurrent neural network (CNN-RNN) framework, enabling precise predictions of corn and soybean yields in 13 states of the US corn belt. Noorunnahar Mst et al. [30] Autoregressive Integrated Moving Average (ARIMA) and Extreme Gradient Boosting (XGBoost) methods were used to predict annual rice production in Bangladesh (1961–2020) and to compare their respective performances. Jiang et al. [31] also employed an LSTM model, leveraging a combination of crop phenology, meteorology, and remote sensing data to forecast maize yield at the county scale. Yuan Liu et al. [32] capitalized on machine learning and deep learning algorithms to devise wheat yield prediction models, leveraging satellite-derived high-resolution and coarse-resolution SIF, vegetation indices, and other pertinent data. Their work comprehensively evaluates and compares the performance of these models.Moreover, the current advancements in machine learning research methodologies facilitate the automatic and cost-effective acquisition of high-accuracy production data [33]. Through the implementation of IoT (Internet of Things) equipment, real-time meteorological data, soil conditions, crop diseases and pests, nutrient deficiencies, and other critical information within the planting area can be captured and transmitted to designated locations via sensors. This approach ensures the collection of high-precision, vast volumes of data, ultimately enhancing the accuracy of predictive models.

A recent scholarly inquiry delves into the integration of machine learning and crop modeling to enhance the accuracy of crop yield forecasting in the US corn belt. The principal objective aims to investigate whether the hybrid approach of crop modeling and machine learning (ML) can yield a superior predictive model, providing the utmost precision in yield projections, and identify the most efficacious crop modeling functions and ML integration strategies for corn yield prediction [34]. Additionally, although efforts have been made in real-time prediction of sugarcane yield based on harvester engine parameters and ML methods [35], there remains a scarcity of studies examining the utilization of diverse ML algorithms to construct an intelligent algorithmic ensemble for predicting crop phenotypic traits and determining crop yield.

In the realm of constructing machine learning models, certain researchers employ diverse algorithms to build identical models and conduct comparative analyses [36]. Virendra Kumar Shrivastava [37] and colleagues conducted research on machine learning (ML) technology, utilizing various input features to forecast the temperature in New Delhi for the next year with a 6-hour resolution. They compared and analyzed the prediction results obtained from the Deep Neural Network Model (DNNM) with those from a multiple regression model, achieving commendable outcomes. Kanchan Bala [38] employed grid search and bagging techniques to optimize the selected classifier (SVM-RBF), ultimately identifying the optimal classifier. However, there is a scarcity of research that combines the construction and comparative analysis of intelligent algorithm model groups utilizing multiple machine learning algorithms with the prediction of crop phenotypic traits based on the characteristics of the research subjectSpecifically, the field of smart agriculture still lacks a comprehensive approach for leveraging phenotypic characteristics of multiple crop varieties to forecast yield and guide cross-breeding strategies. Therefore, further exploration of novel methodologies utilizing various ML techniques to mine phenotypic data for crop yield prediction is imperative.

In the present study, we focused on predicting two critical sugarcane phenotypic characteristics that significantly influence yield: stem diameter and plant height. Initially, we curated a dataset comprising six phenotypic traits from the sugarcane resource nursery at Yunnan Agricultural University. To assess the performance of sugarcane yield prediction, we employed eight diverse machine learning algorithms, encompassing logistic regression, linear regression, K-Nearest Neighbors (KNN), Support Vector Machine (SVM), Backpropagation Neural Network (BPNN), Decision Tree, Random Forest, and XGBoost, to construct predictive models. Our research aims to address the following fundamental inquiries: (1) Which machine learning algorithm, or combination of algorithms, offers superior predictive capabilities for sugarcane phenotypic characteristics? (2) Which intelligent algorithm, or algorithms, is most effective in developing a predictive model that accurately captures the key factors determining yield?

## Study area and data processing

### Study area

The Sugarcane Resources Research Institute at Yunnan Agricultural University conducted a comprehensive survey and collection of wild sugarcane germplasm resources across China spanning the years 1985 to 1993. During this period, they successfully gathered 824 clones encompassing 18 species from 9 distinct genera within the Sugarcane Subrace. Leveraging advanced scientific conservation techniques, the institute established a sugarcane resource garden, where the planted wild sugarcane plants have been iteratively maintained to the present day [39]. These wild varieties have further been utilized in breeding programs, resulting in the development of new sugarcane cultivars through hybridization with existing commercial varieties. In the current study, we have developed an intelligent model group to predict sugarcane yield. This model group was constructed by analyzing phenotypic traits of sugarcane plants in the Yunnan sugarcane resource nursery and integrating machine learning algorithms. The resulting model not only offers a predictive framework but also serves as a platform for further popularization and demonstration, thus contributing to the enhancement of sugarcane cultivation and yield optimization.

### Data set and data preprocessing

#### Datasets

By collecting phenotypic character data from wild sugarcane in Yunnan sugarcane resource nursery and from new sugarcane varieties formed through wild sugarcane hybridization, data support is provided for the construction of yield prediction model based on sugarcane phenotypic character data.

Dataset 1: Data pertaining to 33 phenotypic traits were comprehensively gathered, encompassing species name, plant height, stem diameter, tiller count, underground stem characteristics, internode length, stem color and shape, bud groove presence, wax band presence, node characteristics, root point characteristics, bud shape, bud size, bud color, bud growth status, leaf length, leaf width, widest main vein dimensions, leaf color, leaf tongue characteristics, ear morphology, leaf sheath features, hair group No. 57 of the leaf sheath, flag leaf length and width, inflorescence length, inflorescence maximum width, inflorescence color, inflorescence axis characteristics, growth period, flowering period, fruiting period, and field brix. The initial dataset comprised 1068 wild sugarcane plants, resulting in a data matrix of 1068 rows and 33 columns.

Dataset 2: In a scientific investigation, phenotypic trait data from 572 wild sugarcane plants were systematically collected, encompassing key attributes such as species name, plant height, stem diameter, internode length, leaf length, leaf width, and field brix. The dataset comprised a total of nine phenotypic traits, arranged in a matrix format with 572 rows and nine columns.

#### Data analysis and processing

Initially, dataset 1 and dataset 2 were merged into a unified experimental dataset. Subsequently, an analysis revealed that stem diameter and plant height were the phenotypic traits most strongly correlated with sugarcane yield. Further refinement identified six key phenotypic traits: plant height, stem diameter, internode length, leaf length, leaf width, and field brix, which were selected as predictive indicators of significant research value for wild sugarcane yield, as referenced in previous studies [40, 41]. After rigorous data screening, the principle of outlier handling was applied, resulting in the elimination of data points outside the normal distribution range. The remaining 555 groups of valid data, totaling 3330 data points, are presented in Table 1. The refined experimental dataset, excluding redundant and abnormal data, comprises the aforementioned six phenotypic traits: plant height, stem diameter, internode length, leaf length, leaf width, and field brix, with a dataset size of 555 * 6.

**Table 1:** Collection of experimental data (in cm).

Sample number	Plant height	Stem diameter	Interval length	Leaf length	Leaf width	Field brix(%)
1	13.33	0.30	8.63	92.93	0.93	12.70
2	23.20	0.40	1.30	41.50	0.40	6.00
3	25.67	0.29	12.33	72.87	0.20	15.13
. . .	. . .	. . .	. . .	. . .	. . .	. . .
555	497.00	1.60	30.00	138.00	3.70	7.00

Following the selection of phenotypic characteristics, the dataset was classified in accordance with the Specification for the Description of Sugarcane Germplasm Resources and Data Standards [42], along with the expert advice of the sugarcane research team. Table 2 outlines the categorization rules for the phenotypic traits of wild sugarcane. As shown, plants with a height of 0–99 cm constitute the first category, while 100 cm is designated as the threshold for effective stems. Each subsequent 50 cm increment in plant height represents a new category, totaling nine distinct categories. For stem diameter, the range of 0–0.49 cm comprises the initial category, and each additional 0.2 cm increment signifies a new category, yielding eight categories overall. Internode length is initially classified into the 0–4.9 cm range, with every subsequent 5 cm increment representing a new type, resulting in seven types. Leaf length is initially categorized within 0–4.9 cm, and every 50 cm increment designates a new category, totaling ten categories. Leaf width is initially classified as 0–0.49 cm, with each additional 0.5 cm constituting a new category, resulting in eleven categories. Lastly, field brix is initially categorized as 0–9.99%, and every 2% increment establishes a new category, totaling six categories.

**Table 2:** Classification rule table for phenotypic characteristics of wild sugarcane (in cm).

	Plant height	Stem diameter	Interval length	Leaf length	Leaf width	Field brix (%)
Category 1	0–99	0–0.49	0–4.9	0–49.9	0–0.49	0–9.99
Category 2	100–149	0.50–0.69	5.00–9.9	50–69.9	0.50–0.99	10–11.99
Category 3	150–199	0.70–0.89	10.00–14.9	70–89.9	1.00–1.49	12–13.99
Category 4	200–249	0.90–1.09	15.00–19.9	90–109.9	1.50–1.99	14–15.99
Category 5	250–299	1.10–1.29	20.00–24.9	110–129.9	2.00–2.49	16–17.99
Category 6	300–349	1.30–1.49	25.00–29.9	130–149.9	2.50–2.99	18–19.99
Category 7	350–399	1.50–1.69	30.00–34.9	150–169.9	3.00–3.49	
Category 8	400–449	1.70–1.89		170–189.9	3.50–3.99	
Category 9	450–499			190–209.9	4.00–4.49	
Category 10				210–229.9	4.50–4.99	
Category 11					5.00–5.49	

The original data was systematically classified in accordance with the categorization rules outlined in Table 2. Post-classification, the data was consolidated into a unified dataset, resulting in the compilation presented in Table 3. Notably, following the application of these rules, the dataset size expanded to 555 * 12, reflecting the integration of both the raw data and the categorical information.

**Table 3:** Raw data classification data table (in cm).

Samsam number	Plant height class	Plant height	Stem diameter class	Stem diameter	Interval length class	Interval length	Leaf length class	Leaf length	Leaf width	Leaf width	Field brix class	Field brix(%)
1	Category 1	13.33	Category 1	0.30	Category 2	8.63	Category 4	92.93	Category 2	0.93	Category 4	12.70
2	Category 1	23.20	Category 1	0.40	Category 1	1.30	Category 1	41.50	Category 1	0.40	Category 1	6.00
3	Category 1	25.67	Category 1	0.29	Category 3	12.33	Category 3	72.87	Category 1	0.20	Category 4	15.13
. . .	. . .	. . .	. . .	. . .	. . .	. . .	. . .	. . .	. . .	. . .	. . .	. . .
555	Category 9	497.00	Category 7	1.60	Category 7	30.00	Category 6	138.00	Category 8	3.70	Category 1	7.00

Data standardization is the process of scaling data to a predefined range, effectively eliminating the constraints of units and transforming it into a dimensionless pure value. This approach enables the comparison and weighting of indicators with varying units or magnitudes.

A prime example of data standardization is data normalization, which transforms the raw data into a decimal range between (0,1). In the current study, we employ the Min-Max standardization algorithm [43] as the preferred method for data standardization.

Specifically, the standardized processing of data involves the following steps:

Transform sequence *x*_1_, *x*_2_,⋯,*x*_*n*_:

$$y_{i}=\frac{x_{i}-\underset{\begin{array}{c} 1⩽j⩽n \end{array}}{min}x_{j}}{\underset{\begin{array}{c} 1⩽j⩽n \end{array}}{max}x_{j}-\underset{\begin{array}{c} 1⩽j⩽n \end{array}}{min}x_{j}}$$ (1)

Get new sequence *y*_1_, *y*_2_,⋯,*y*_*n*_∈[0,1]. In this study, the Min-Max standardization method was employed to execute a linear transformation on the original dataset, subsequently mapping the values to the range of [0,1]. Concurrently, several phenomena within the dataset were identified, and these were efficiently addressed through the application of random oversampling techniques.

After performing random oversampling with stem diameter and plant height as dependent variables, the data are shown in Table 4. The sample balance of random oversampling data with stem diameter (plant height) as dependent variable is 12.5% (11.1%) of each category. To avoid any potential impact on modeling accuracy due to the oversampling of certain parts of the sample data, SMOTE-ENN combined sampling is performed for data equalization again. After standardization, the SMOTE-ENN combination sampling was conducted again after the random oversampling with stem diameter and plant height as the dependent variables. Consequently, the data volume after the random oversampling process with plant height (stem diameter) as the dependent variable reached 1620 * 12 (1151 * 12).

**Table 4:** Experimental data set.

Implicit variable	Samsam number	Stem diameter(cm)	Random oversampling	Smote-enn combination sampling	Plant height(cm)	Random oversampling	SMOTE-ENN combination sampling
**Stem diameter**	1	0.37	0.057915058	0.156370656	28.33333333	0.028945555	0.031013094
	2	0.303333333	0.037904893	0.137065637	31.66666667	0.117760618	0.043418332
	3	0.656666667	0.322393822	0.028957529	32.00	0.038594073	0.044107512
	4	0.31	0.121621622	0.063706564	29.00	0.032391454	0.035837354
	5	0.3075	0.120173745	0.106177606	35.33333333	0.045485872	0.05444521
	. . .	. . .	. . .	. . .	. . .	. . .	. . .
**Sample size**	555		1768	1390		1768	1390
**Plant height**	1	0.546666667	0.258687259	0.258687259	30.66666667	0.035837354	0.035837354
	2	0.40	0.173745174	0.057915058	23.20	0.020399724	0.026188835
	3	0.29	0.11003861	0.057915058	25.66666667	0.025499655	0.028945555
	4	0.31	0.121621622	0.120173745	29.00	0.032391454	0.045485872
	5	0.3075	0.120173745	0.113899614	35.33333333	0.045485872	0.066161268
	. . .	. . .	. . .	. . .	. . .	. . .	. . .
**Sample size**	555		1620	1511		1620	1511

## Methods

Due to the close relationship between sugarcane yield and key phenotypic traits, specifically the direct proportionality between plant height and stem diameter values and yield, it is evident that a higher plant height and larger stem diameter indicate a greater weight per plant and hence higher yield. Therefore, constructing a sugarcane plant height/stem diameter prediction model based on machine learning algorithms enables accurate prediction of plant height/stem diameter values, given the collection of other important phenotypic traits of sugarcane. This, in turn, indirectly estimates the yield potential of sugarcane. Additionally, in the process of sugarcane hybrid breeding, the key phenotypic trait prediction model developed in this study, which can precisely predict plant height/stem diameter values, can provide materials for the artificial selection of new sugarcane varieties with superior stem diameter and plant height, thus filling the research gap in indirectly predicting yield through phenotypic traits of sugarcane.

This study integrates various machine learning algorithms, encompassing both non-integrated and integrated learning approaches, to construct an intelligent predictive model for wild sugarcane. The research delves into the analysis of the prediction outcomes and model accuracy, thereby investigating the significance of employing machine learning methodologies in forecasting phenotypic traits of wild sugarcane.

### Algorithm selection

In this study, we utilized six distinct non-integrated learning algorithms, namely decision tree, multiclass logistic regression, K-nearest neighbors (KNN), support vector machine (SVM), backpropagation neural network (BPNN), and linear regression. Additionally, we employed two integrated learning algorithms: random forest and XGBoost [44]. The aim was to comprehensively compare and analyze the performance of these algorithms.

### Setting of evaluation indicators

In the classification experiment, we employ accuracy, recall, precision, and F1-score as metrics to evaluate the performance of the classification model. Similarly, in the regression experiment, mean squared error (MSE), root mean squared error (RMSE), mean absolute error (MAE), and coefficient of determination (R^2^) are used as the evaluation indicators [45]. The characteristics of these metrics are outlined below:

The confusion matrix [46] serves as a matrix representation of the classification model’s prediction outcomes. It is a valuable tool for evaluating the model’s classification performance. Specifically, the columns of the confusion matrix represent the predicted values, while the rows correspond to the actual values. Each element within the matrix quantifies the frequency of instances where the classifier’s prediction aligns with the true category. The confusion matrix facilitates the calculation of accuracy, recall, precision, and F1-score.


$$Accuracy=\frac{TP+TN}{TP+TN+FP+FN}$$ (2)

$$Precision=\frac{TP}{TP+FP}$$ (3)

Recall rate: in the results that are actually positive samples, the predicted proportion is positive samples. The larger the recall rate, the better.


$$Recall=\frac{TP}{TP+FN}$$ (4)

F1: The harmonic average of precision and recall. Precision and recall are interdependent, and although both values are high, it is an ideal situation. However, in reality, it is often a case of high precision and low recall, or low recall but high precision. If it is necessary to balance both, then the F1 indicator can be used.


$$F1=2\times \frac{Precision\times Recall}{Precision+Recall}$$ (5)

MSE (Mean Square Error): The expected value of the square of the difference between the predicted value and the actual value. The smaller the value, the higher the accuracy of the model.


$$MSE=\frac{1}{n}\sum_{i=1}^{n}{\left(y_{i}-{\hat{y}}_{i}\right)}^{2}$$ (6)

RMSE (Root Mean Square Error): is the square root of MSE, and the smaller the value, the higher the accuracy of the model.


$$\mathrm{RMSE}=\sqrt{\frac{1}{n}\sum_{i=1}^{n}{\left(y_{i}-{\hat{y}}_{i}\right)}^{2}}$$ (7)

MAE (Mean Absolute Error): The average value of absolute error, which can reflect the actual situation of prediction error. The smaller the value, the higher the accuracy of the model.


$$MAE=\frac{1}{n}\sum_{i=1}^{n}|y_{i}-{\hat{y}}_{i}|$$ (8)

R ^2^: Compared to using only the mean, the closer the result is to 1, the higher the accuracy of the model.


$$R^{2}=1-\frac{\sum_{i=1}^{n}(y_{i}-{\hat{y}}_{i}{)}^{2}}{\sum_{i=1}^{n}(y_{i}-{\overset{¯}{y}}_{i}{)}^{2}}$$ (9)

### Experimental design

In this comprehensive study, we have primarily established a comprehensive data resource library through diligent collection, systematic organization, and rigorous analysis of data pertaining to sugarcane phenotypic traits. Leveraging the distinctive features of this data resource library, we have selected eight distinct machine learning algorithms, each exhibiting varied performance, to develop predictive models for two crucial phenotypic characteristics: stem diameter and plant height. These characteristics serve as fundamental determinants of sugarcane yield, ultimately aiming to attain the objective of predicting sugarcane yield, particularly in high-yield scenarios. The subsequent sections detail the experimental design.

In our experimental setup, we initially utilized the standardized 555 sets of data alongside randomly oversampled data comprising 1620 plant heights and 1768 stem diameters, which were designated as the testing and training sets. Subsequently, we employed eight diverse algorithms to classify and regress the plant height and stem diameter separately. For the classification prediction model, we adopted seven algorithms, namely decision tree, multiclass logistic regression, KNN, support vector machine, BP neural network, random forest, and XGBoost. However, when constructing the regression prediction model, we substituted multiclass logistic regression with linear regression and utilized algorithms such as CART decision tree, linear regression, KNN, support vector machine, BP neural network, random forest, and XGBoost. During the experiment, we compared the efficacy of ensemble learning with non-ensemble learning approaches and analyzed the model accuracy before and after random oversampling. Notably, during the modeling process, 70% of the data was allocated for training, while 30% was designated for testing. When utilizing each model, the parameter adjustments were tailored to match the corresponding classification model parameters. Overall, a total of 84 experiments were conducted, and the performance of each experimental model was evaluated based on its stable value, which was deemed as the final result.

**Fig 1:**
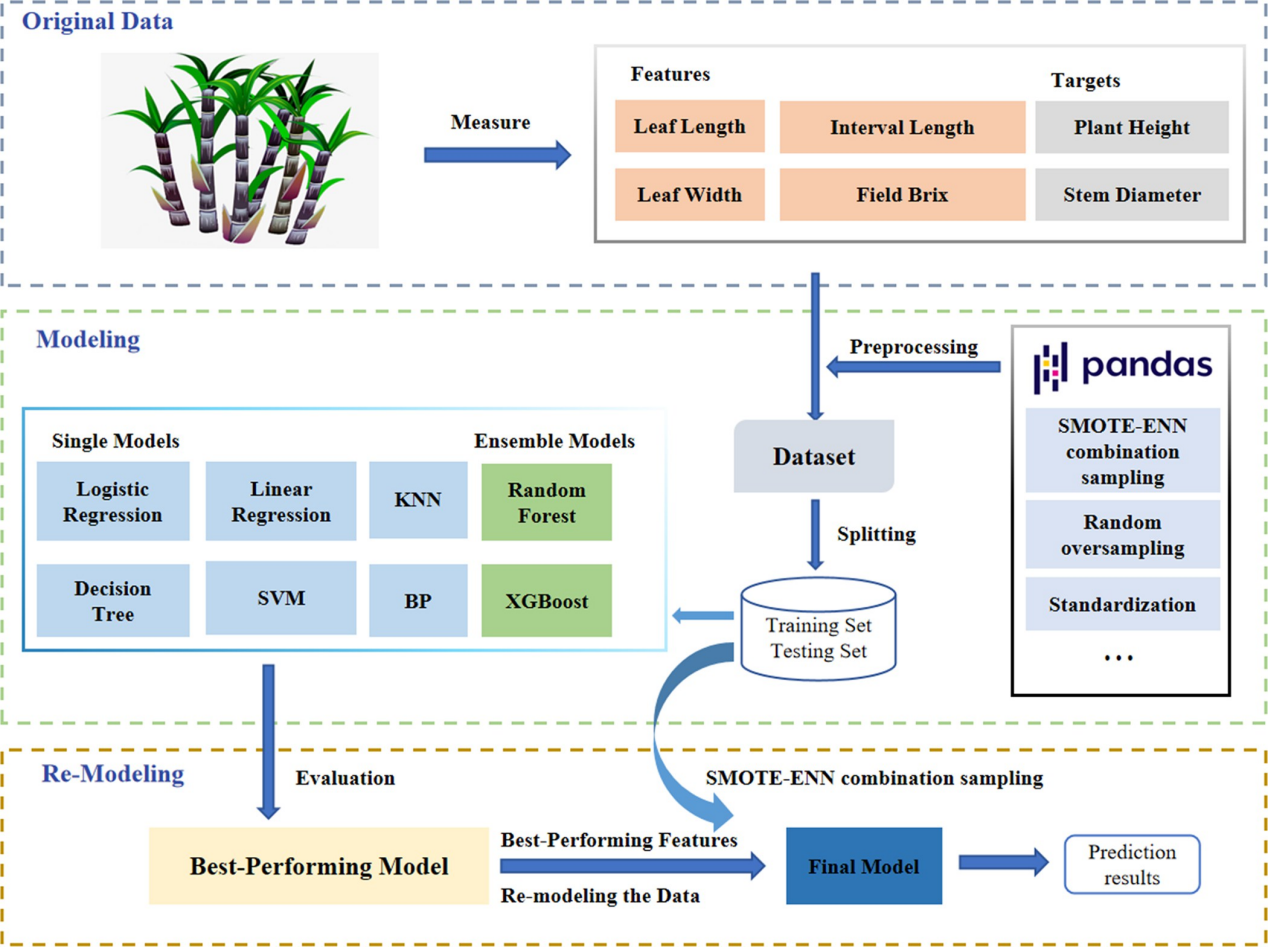
Flowchart for modeling important phenotypic characters in sugarcane.

By employing a self-generated data integration pattern, we successfully augmented the dataset. Specifically, we appended modified values of various phenotypic traits: (plant height + 100 * 0.4), (stem diameter + 0.2 * 0.4), (internode length + 5 * 0.4), (leaf length + 20 * 0.4), (leaf width + 0.5 * 0.4), and (field brix + 2 * 0.4). The randomly oversampled data were then utilized for machine learning modeling.Upon acquiring the initial results, we implemented SMOTE-ENN combination sampling to model the prediction outcomes in a similar manner. This step allowed us to compare the performance of different modeling approaches. Fig 1 illustrates the experimental design process, outlining the integration of the self-generated data pattern, data augmentation, modeling, and the application of SMOTE-ENN combination sampling.

## Results

### A predictive model for key phenotypic traits of sugarcane, based on machine learning, has been constructed

This model primarily utilizes seven phenotypic traits of sugarcane, including plant height, stem diameter, third node length, internode length, leaf length, leaf width, and field brix, as the dataset for constructing predictive models of key phenotypic traits (plant height and stem diameter). Five to eight suitable machine learning algorithms are selected based on data characteristics such as the volume and size of the collected data. Taking the six phenotypic traits of stem diameter, third node length, internode length, leaf length, leaf width, and field brix as influencing factors, an intelligent predictive model is constructed for plant height prediction. The performance of different machine learning models is compared, and the model with the best performance is selected as the predictive model for sugarcane plant height. Similarly, a predictive model for sugarcane stem diameter is constructed using the same method.

### Performance comparison of sugarcane stem diameter/plant height classification prediction models using different machine learning algorithms

**Fig 2:**
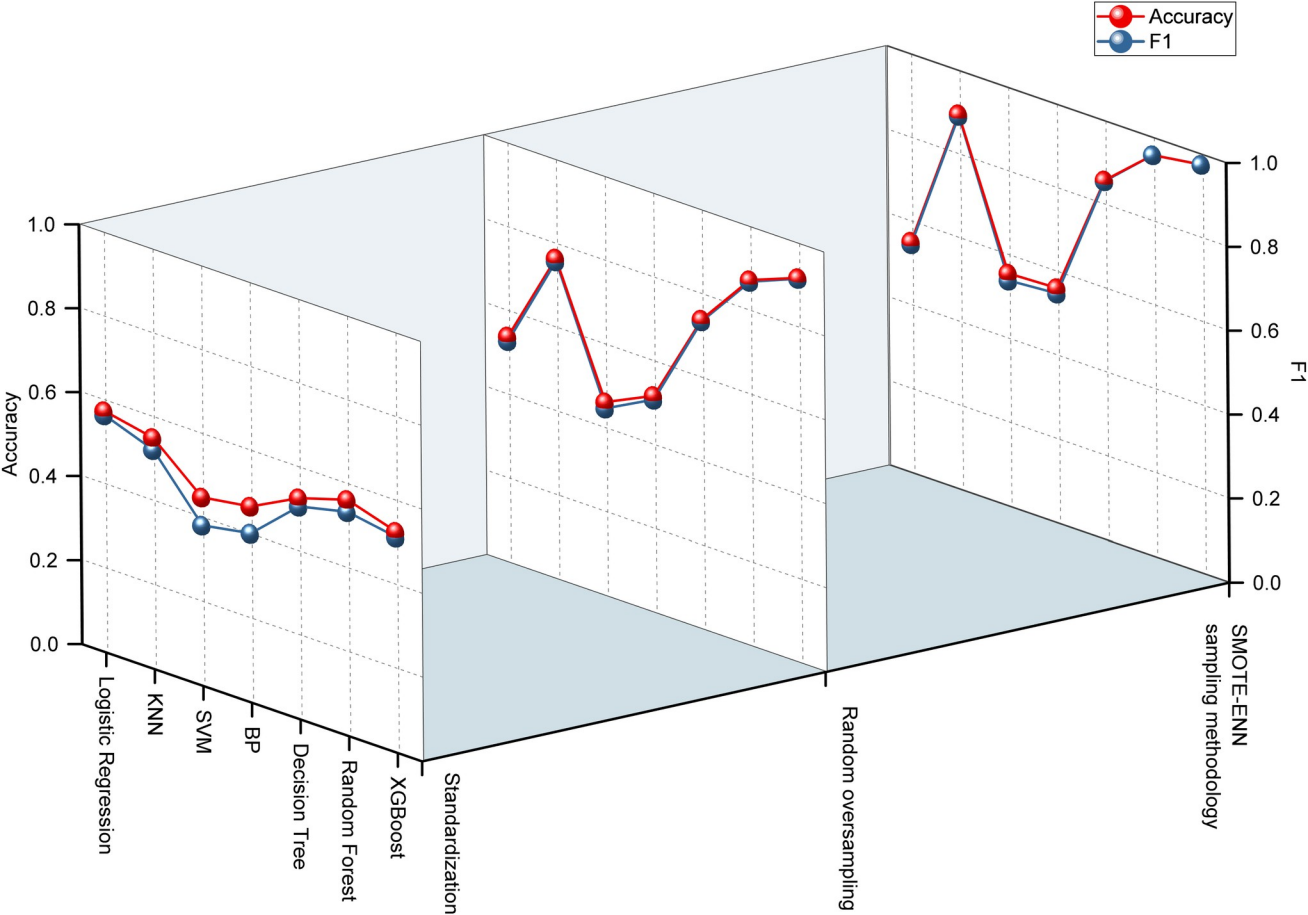
Comparison of the performance of sugarcane stem diameter classification prediction models.

In the experiment aimed at predicting stem diameter classification, we employed seven algorithms: logistic regression, KNN, support vector machine, BP neural network, decision tree, random forest, and XGBoost, to construct stem diameter classification prediction models. The outcomes of these experiments are presented in Table 5. Following data standardization, the stem diameter was directly modeled for prediction experiments. Among the various algorithms, logistic regression exhibited the highest modeling accuracy, with evaluation index values spanning from 0.56 to 0.59. Conversely, the support vector machine algorithm displayed the lowest accuracy, maintaining values between 0.33 and 0.45. After random oversampling, the performance of the algorithms changed significantly. The XGBoost model achieved the highest modeling accuracy, with evaluation index values ranging from 0.91 to 0.92. However, the support vector machine continued to exhibit the lowest accuracy, maintaining values between 0.44 and 0.47. Logistic regression and the BP neural network performed similarly, while KNN and the decision tree also showed comparable accuracies.When utilizing SMOTE-ENN combined sampling for modeling, the XGBoost and Random Forest algorithms displayed high modeling accuracy, with various evaluation indicators stabilizing between 0.97 and 0.98. Conversely, the modeling accuracy of the support vector machine, logistic regression, and BP neural network algorithms remained relatively low, ranging from 0.54 to 0.57. The other models performed well. Fig 2 provides a comparative analysis of the modeling accuracy of the seven algorithms under different data processing methods, using F1 and Acc indicators. The results indicate that random oversampling can improve model accuracy to a certain extent, while the SMOTE-ENN combined sampling method can comprehensively enhance model performance. Furthermore, the XGBoost and Random Forest algorithms emerged as the most effective for sugarcane stem diameter classification modeling.

**Table 5:** Stem diameter/plant height classification prediction model accuracy table.

Implicit variable	Data Manipulate	Evaluation Norm	Logistic regression	Knn	Svm	Bp	Decision tree	Random forest	Xgboost
**Stem diameter**	**Standardization**	Accuracy	0.575	0.551	0.449	0.467	0.527	0.563	0.527
		Recall	575	0.551	0.449	0.467	0.527	0.563	0.527
		Precision	0.589	0.518	0.337	0.359	0.497	0.533	0.519
		F1	0.565	0.522	0.382	0.403	0.507	0.534	0.514
	**Random oversampling**	Accuracy	0.54	0.766	0.463	0.518	0.74	0.874	0.919
		Recall	0.54	0.766	0.463	0.518	0.74	0.874	0.919
		Precision	0.537	0.757	0.466	0.514	0.74	0.874	0.918
		F1	0.529	0.759	0.448	0.509	0.736	0.87	0.917
	**SMOTE-ENN sampling methodology**	Accuracy	0.553	0.897	0.557	0.562	0.859	0.959	0.976
		Recall	0.553	0.897	0.557	0.562	0.859	0.959	0.976
		Precision	0.57	0.9	0.553	0.555	0.862	0.963	0.977
		F1	0.547	0.893	0.54	0.549	0.857	0.959	0.976
**Plant height**	**Standardization**	Accuracy	0.419	0.425	0.425	0.419	0.359	0.485	0.497
		Recall	0.419	0.425	0.425	0.419	0.359	0.485	0.497
		Precision	0.424	0.384	0.386	0.394	0.322	0.454	0.496
		F1	0.397	0.392	0.402	0.401	0.336	0.466	0.493
	**Random oversampling**	Accuracy	0.671	0.722	0.588	0.63	0.73	0.827	0.864
		Recall	0.671	0.722	0.588	0.63	0.73	0.827	0.864
		Precision	0.64	0.705	0.586	0.601	0.745	0.812	0.861
		F1	0.646	0.709	0.576	0.598	0.729	0.816	0.861
	**SMOTE-ENN sampling methodology**	Accuracy	0.847	0.939	0.789	0.777	0.936	0.974	0.974
		Recall	0.847	0.939	0.789	0.777	0.936	0.974	0.974
		Precision	0.845	0.941	0.764	0.74	0.929	0.976	0.974
		F1	0.839	0.939	0.764	0.749	0.928	0.972	0.973

**Fig 3:**
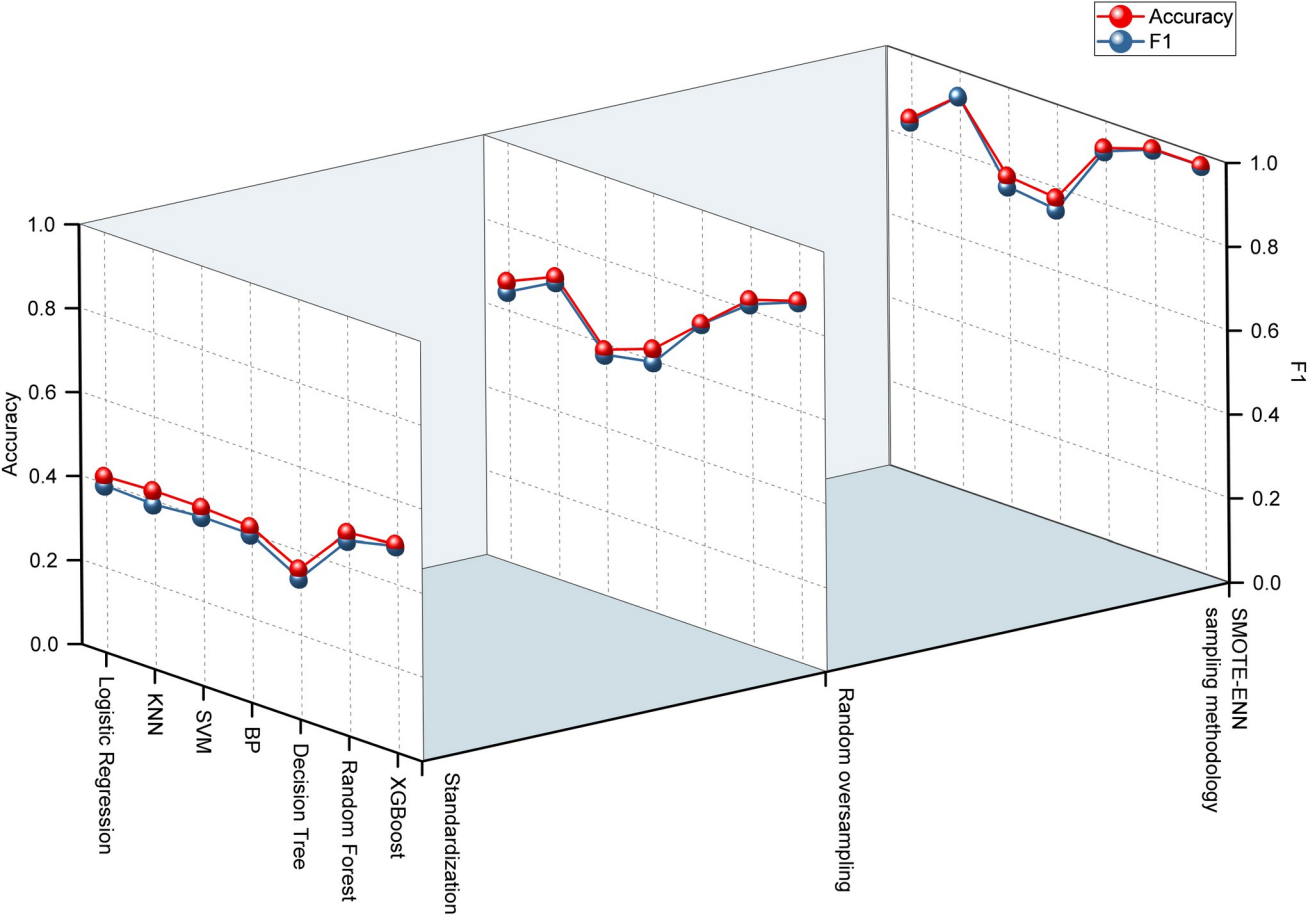
Comparison of the performance of sugarcane plant height classification prediction models.

The experiment conducted for plant height classification prediction adhered to a methodology analogous to the aforementioned approach in constructing a predictive model. As evident in Table 5, post-data standardization, the XGBoost and Random Forest algorithms exhibited remarkable accuracy in forecasting plant height, with evaluation metrics ranging between 0.45 and 0.50, surpassing other models that registered approximately 0.40 in accuracy. Upon the application of random oversampling, a substantial enhancement in modeling precision was observed across all models. Specifically, the XGBoost and Random Forest algorithms led the way, achieving evaluation index values spanning from 0.81 to 0.87. Similarly, the KNN and decision tree algorithms also witnessed significant improvements, reaching accuracy levels of 0.73 and 0.75 respectively. Other models maintained a stable accuracy of approximately 0.6.Utilizing the combined SMOTE-ENN sampling methodology, each model attained its peak accuracy. Notably, the KNN, decision tree, XGBoost, and Random Forest algorithms all exceeded the 0.9 accuracy threshold. Meanwhile, other algorithms attained an accuracy of approximately 0.75. Fig 3 offers a comparative analysis of the modeling accuracy of seven algorithms, employing F1 and Acc metrics, under various data processing techniques. The results unequivocally demonstrate that the SMOTE-ENN combined sampling method significantly enhances model accuracy, with the XGBoost and Random Forest algorithms emerging as the most effective in sugarcane height classification modeling.

### Performance comparison of sugarcane stem diameter/plant height regression prediction models using different machine learning algorithms

**Fig 4:**
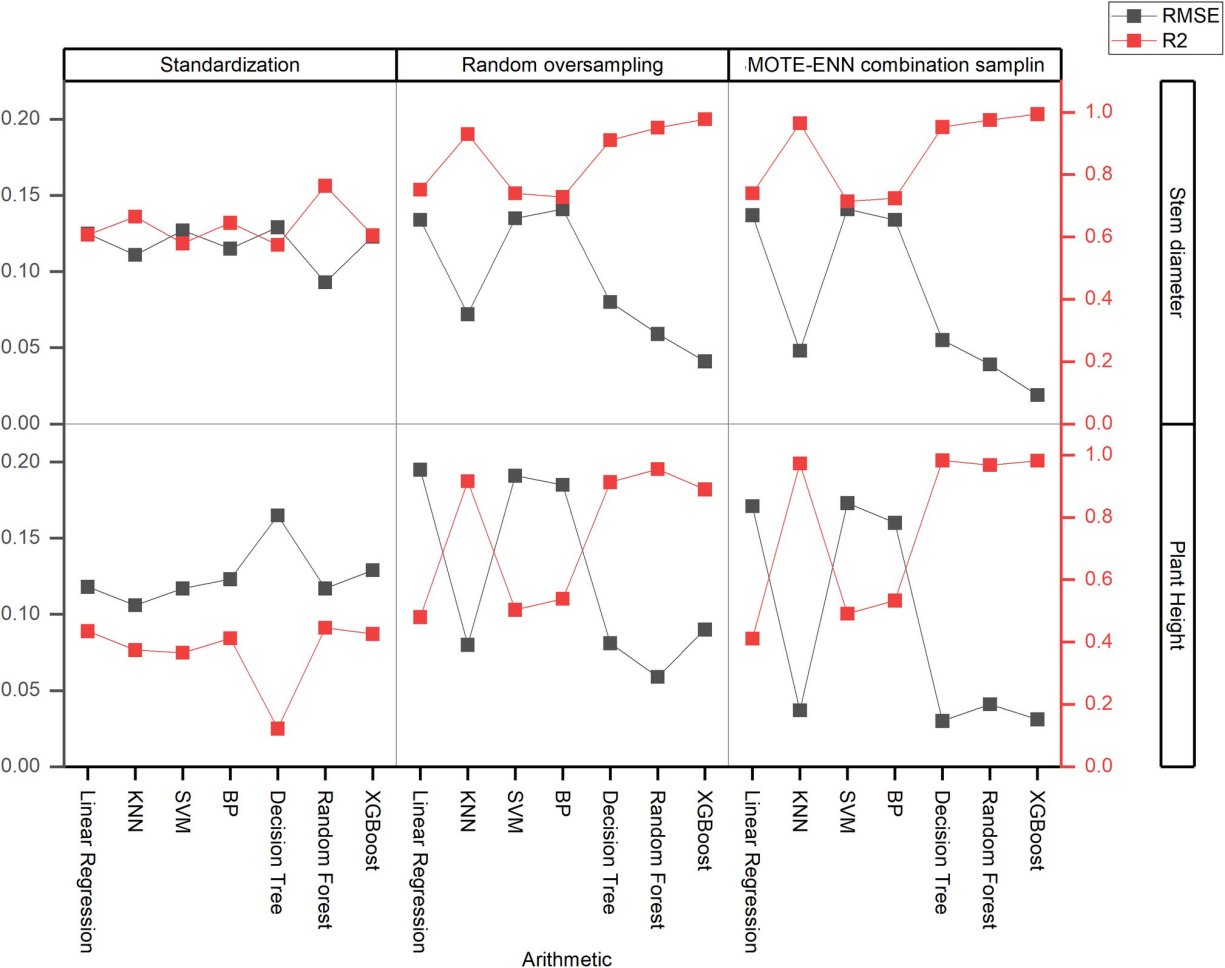
Comparison of the performance of sugarcane stem diameter/plant height regression prediction models.

In the experiment aimed at stem diameter and plant height regression prediction, seven algorithms were employed to establish sugarcane stem diameter prediction models. These encompassed linear regression, KNN, support vector machine, backpropagation (BP), decision tree, random forest, and XGBoost. The study constructed the models by applying various data processing techniques, including data standardization, random oversampling, and combined sampling. The performance of these models was rigorously evaluated using metrics such as MSE, RMSE, MAE, and R2. The comprehensive results of this analysis are presented in Table 6, which offers a detailed performance analysis of the 42 models constructed using the seven algorithms. Furthermore, Fig 4 provides a comparative analysis of the modeling accuracy of the seven algorithms under different data processing methods, utilizing RMSE and R2 as the primary indicators. Notably, the decision tree, random forest, and XGBoost algorithms exhibited superior performance in model construction, with the XGBoost algorithm standing out as the most effective in constructing stem diameter regression prediction models.

**Table 6:** Stem diameter/plant height regression prediction model accuracy table.

Implicit variable	Data manipulate	Evaluation Norm	Linear regression	KNN	SVM	BP	Decision tree	Random forest	XGBoost
**Stem diameter**	**Standardization**	MSE	0.016	0.012	0.016	0.013	0.017	0.009	0.015
		RMSE	0.125	0.111	0.127	0.115	0.129	0.093	0.123
		MAE	0.093	0.081	0.093	0.086	0.095	0.074	0.09
		R^2^	0.607	0.665	0.579	0.645	0.575	0.764	0.605
	**Random oversampling**	MSE	0.018	0.005	0.018	0.02	0.006	0.003	0.002
		RMSE	0.134	0.072	0.135	0.141	0.08	0.059	0.041
		MAE	0.104	0.039	0.105	0.109	0.051	0.041	0.014
		R^2^	0.752	0.93	0.74	0.728	0.911	0.951	0.978
	**SMOTE-ENN combination sampling**	MSE	0.019	0.002	0.02	0.018	0.003	0.002	0
		RMSE	0.137	0.048	0.141	0.134	0.055	0.039	0.019
		MAE	0.107	0.024	0.113	0.105	0.03	0.024	0.005
		R^2^	0.74	0.965	0.714	0.724	0.953	0.975	0.994
**Plant Height**	**Standardization**	MSE	0.014	0.011	0.014	0.015	0.027	0.014	0.017
		RMSE	0.118	0.106	0.117	0.123	0.165	0.117	0.129
		MAE	0.087	0.082	0.084	0.088	0.11	0.078	0.088
		R^2^	0.435	0.374	0.366	0.412	0.122	0.446	0.426
	**Random oversampling**	MSE	0.038	0.006	0.036	0.034	0.007	0.003	0.008
		RMSE	0.195	0.08	0.191	0.185	0.081	0.059	0.09
		MAE	0.155	0.039	0.148	0.149	0.042	0.034	0.029
		R^2^	0.48	0.916	0.503	0.539	0.913	0.955	0.89
	**SMOTE-ENN combination sampling**	MSE	0.029	0.001	0.03	0.026	0.001	0.002	0.001
		RMSE	0.171	0.037	0.173	0.16	0.03	0.041	0.031
		MAE	0.138	0.014	0.137	0.129	0.012	0.011	0.007
		R^2^	0.411	0.973	0.491	0.533	0.983	0.968	0.982

### Expanding experimental data to construct predictive models

**Fig 5:**
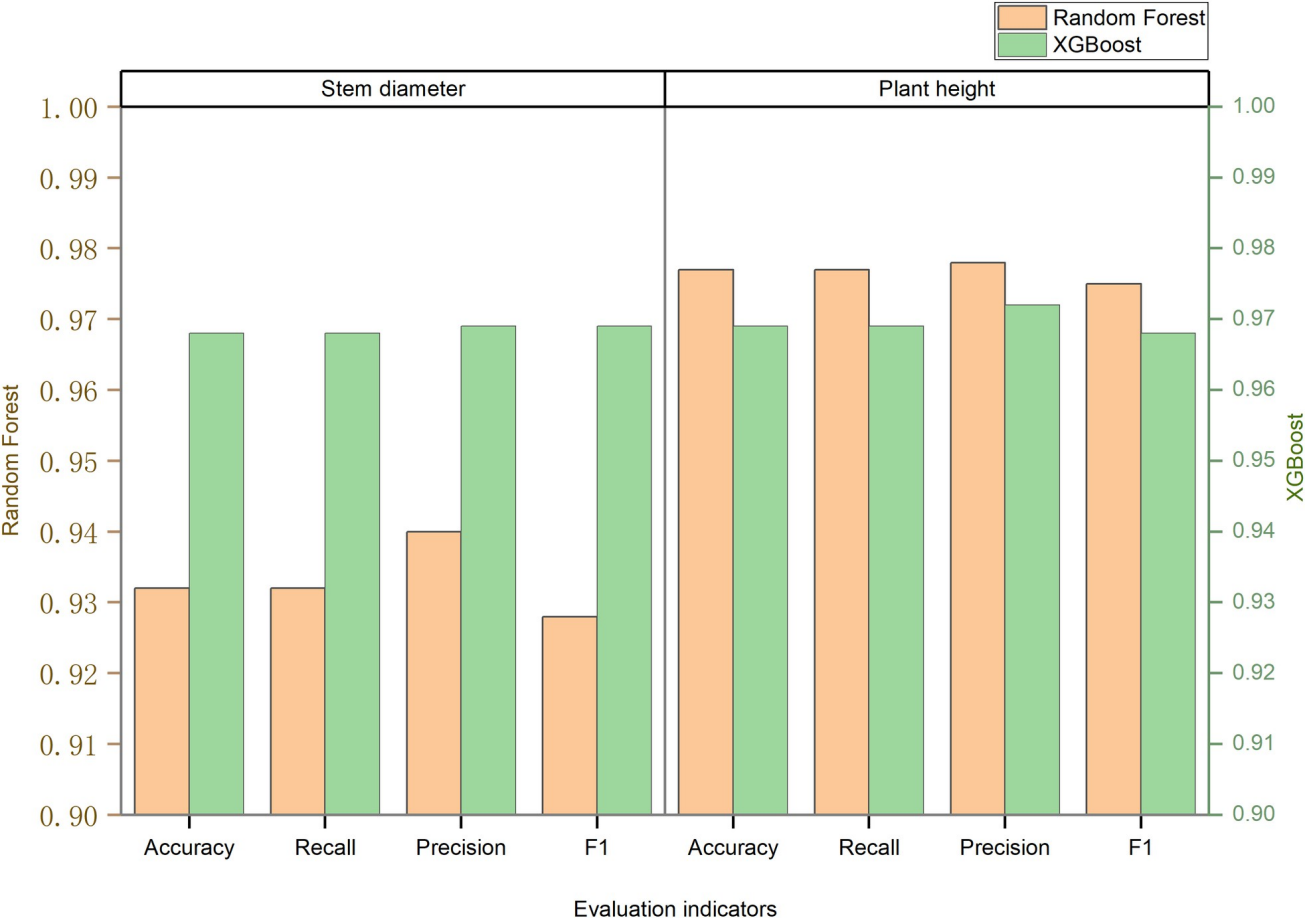
Performance graph of classification model after expanding data.

**Fig 6:**
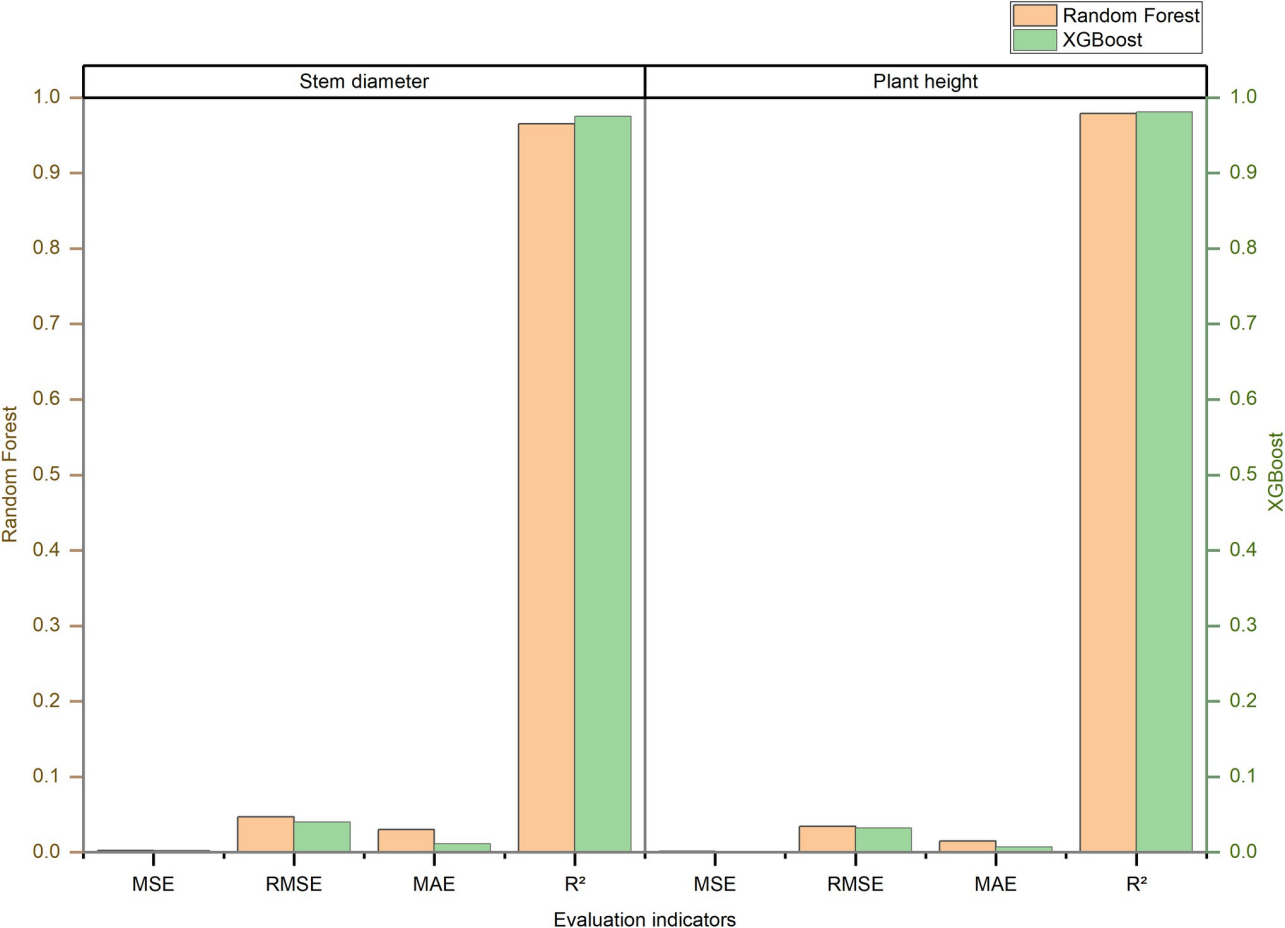
Plot of regression model performance after expanding data.

Initially, a random selection of 100 sets of data was made from the experimental dataset, and the interval distance between the characteristic values of each phenotypic trait was determined. Subsequently, the original data values were augmented by adding a value equivalent to 1.4 times the calculated interval distance, thereby generating 100 novel datasets. These self-generated datasets were then incorporated into the original dataset to form an expanded dataset. Utilizing the optimal modeling algorithms identified, namely Random Forest and XGBoost, modeling was performed on this expanded dataset. As depicted in Fig 5, following the application of SMOTE-ENN combined sampling, the evaluation metrics of the Random Forest algorithm post-modeling ranged from 0.932 to 0.94, indicating a slight decrement in accuracy compared to pre-expansion. Conversely, the XGBoost algorithm exhibited superior performance, with evaluation metrics ranging from 0.968 to 0.969, albeit slightly lower than the pre-expansion accuracy. In the experiment pertaining to plant height classification prediction, the utilization of SMOTE-ENN combined sampling coupled with Random Forest algorithm modeling yielded evaluation metrics spanning from 0.975 to 0.978, representing a marginal improvement over pre-expansion accuracy. However, the XGBoost algorithm exhibited a slight decrement in performance post-modeling, with evaluation metrics ranging from 0.969 to 0.972. In examining the experimental results presented in Table 7 it was observed that the variance in the model evaluation index values, both improvement and decrease, remained approximately 0.005, regardless of the inclusion of self-generated data. Upon incorporating additional experimental data, regression models were constructed using Random Forest and XGBoost algorithms, coupled with SMOTE-ENN sampling, to forecast stem diameter and plant height. As illustrated in Fig 6, both models exhibited commendable performance, with R2 values close to 1 and other metrics not surpassing 0.1. However, a comparative analysis revealed that, compared to models constructed prior to the incorporation of self-generated data, the performance was marginally inferior to that achieved before the expansion of experimental data, as evidenced in Table 7. This suggests that the inclusion of self-generated data in this experiment had a negligible impact on the evaluation index values of the models.

**Table 7:** Comparison table of model performance before and after data expansion.

Categories	Arithmetic	Traits	Expanded data	Accuracy	Recall	Precision	F1
**Classification**	Random Forest	Stem diameter	No data expansion	Recall	0.959	0.963	0.959
			Expanded Data	Precision	0.932	0.94	0.928
		Plant height	No data expansion	0.974	0.974	0.976	0.972
			Expanded Data	0.977	0.977	0.978	0.975
	XGBoost	Stem diameter	No data expansion	0.976	0.976	0.977	0.976
			Expanded Data	0.968	0.968	0.969	0.969
		Plant height	No data expansion	0.974	0.974	0.974	0.973
			Expanded Data	0.969	0.969	0.972	0.968
**Categories**	**Arithmetic**	**Traits**	**Expanded data**	**MSE**	**RMSE**	**MAE**	**R^2^**
**Regression**	Random Forest	Stem diameter	No data expansion	0.002	0.039	0.024	0.975
			Expanded Data	0.002	0.047	0.03	0.965
		Plant height	No data expansion	0.002	0.041	0.011	0.968
			Expanded Data	0.001	0.034	0.015	0.979
	XGBoost	Stem diameter	No data expansion	0	0.019	0.005	0.994
			Expanded Data	0.002	0.04	0.011	0.975
		Plant height	No data expansion	0.001	0.031	0.007	0.982
			Expanded Data	0.001	0.032	0.007	0.981

### The impact of important phenotypic features on the performance of intelligent models

In this study, four types of variables were excluded: "leaf length," "leaf width," "internode length," and "field brix." Subsequently, the SMOTE-ENN combination sampling method was employed for data preprocessing. Utilizing the optimal XGBoost algorithm identified for predicting sugarcane phenotype characteristics, classification and regression models were constructed. Detailed results are presented in Table 8.

**Table 8:** Comparison table of model performance after removing a single influencing factor.

Implicit variable	Manipulate	Classification				Regression			
		Accuracy	Recall	Precision	F1	MSE	RMSE	MAE	R^2^
Plant height	Uncensored data	0.974	0.974	0.974	0.973	0.001	0.031	0.007	0.982
	Reduced leaf length	0.988	0.988	0.989	0.987	0.001	0.029	0.005	0.984
	Reduced leaf width	0.98	0.98	0.98	0.98	0.001	0.023	0.005	0.991
	Reduced internode length	0.974	0.974	0.975	0.973	0.001	0.034	0.007	0.977
	Reduced field brix	0.983	0.983	0.983	0.982	0	0.019	0.005	0.993
Stem diameter	Uncensored data	0.976	0.976	0.977	0.976	0	0.019	0.005	0.094
	Reduced leaf length	0.964	0.964	0.965	0.964	0.001	0.028	0.007	0.988
	Reduced leaf width	0.945	0.945	0.944	0.944	0.002	0.047	0.01	0.966
	Reduced internode length	0.986	0.986	0.986	0.985	0.001	0.03	0.009	0.985
	Reduced field brix	0.974	0.974	0.975	0.974	0.001	0.028	0.008	0.988

### Performance analysis of important phenotypic characteristics on sugarcane stem diameter/plant height classification prediction models

Following the elimination of the "leaf width" variable, both random forest and XGBoost algorithms were implemented to develop stem diameter prediction models. Notably, all evaluation metrics ranged from 0.934 to 0.964. Nevertheless, a comparative analysis with the corresponding models revealed a minor decrement in these evaluation metrics. Specifically, the XGBoost modeling exhibited a 0.011–0.012 decline in various evaluation indicators, while the random forest algorithm modeling saw a 0.21–0.25 reduction.

Subsequently, upon eliminating the "leaf width" variable, XGBoost was employed to construct a plant height prediction model. This model achieved evaluation metrics ranging from 0.976 to 0.976, slightly surpassing the modeling performance. Furthermore, upon the removal of the "internode length" variable, the random forest algorithm exhibited commendable modeling performance, with all evaluation metrics spanning 0.806 to 0.813, albeit slightly inferior to the results. Finally, upon excluding the "field brix" variable and utilizing the random forest algorithm for modeling, all indicators were found to be within the range of 0.975 to 0.978, resulting in superior modeling performance.

### Performance analysis of regression prediction models for sugarcane stem diameter/plant height based on important phenotypic characteristics

In this study, four variables were excluded: "leaf length," "leaf width," "internode length," and "field brix." We employed the SMOTE-ENN combination sampling data processing method and integrated the XGBoost algorithm, which was identified as the optimal model, to construct classification and regression models for predicting sugarcane phenotype characteristics. For a detailed overview, refer to Table 8.

Following the elimination of each influencing factor individually, a classification prediction model for sugarcane plant height was formulated. The experimental results revealed a slight improvement in the accuracy of each indicator, ranging between 0.001 and 0.15, indicating a minimal impact on model performance. Notably, the removal of the "leaf length" factor resulted in the most significant enhancement in model accuracy, suggesting that this factor is redundant and should be excluded. Conversely, the exclusion of the "internode length" factor led to the most significant decrement in model accuracy, implying its pivotal role in predicting plant height. Utilizing a similar research approach, notable variations in the accuracy of the stem diameter classification model were observed. Specifically, eliminating the "leaf width" factor caused the most significant deterioration in model performance, whereas the exclusion of the "internode length" factor led to the greatest improvement. This finding suggests that leaf width is a crucial factor in the model, while internode length is not a significant influencing factor and should be excluded.When developing a regression prediction model for plant height, the removal of the "field brix" factor resulted in the most significant decrement in model performance, indicating its importance in the model. The elimination of other factors led to relatively minor changes in various evaluation metrics.

**Fig 7:**
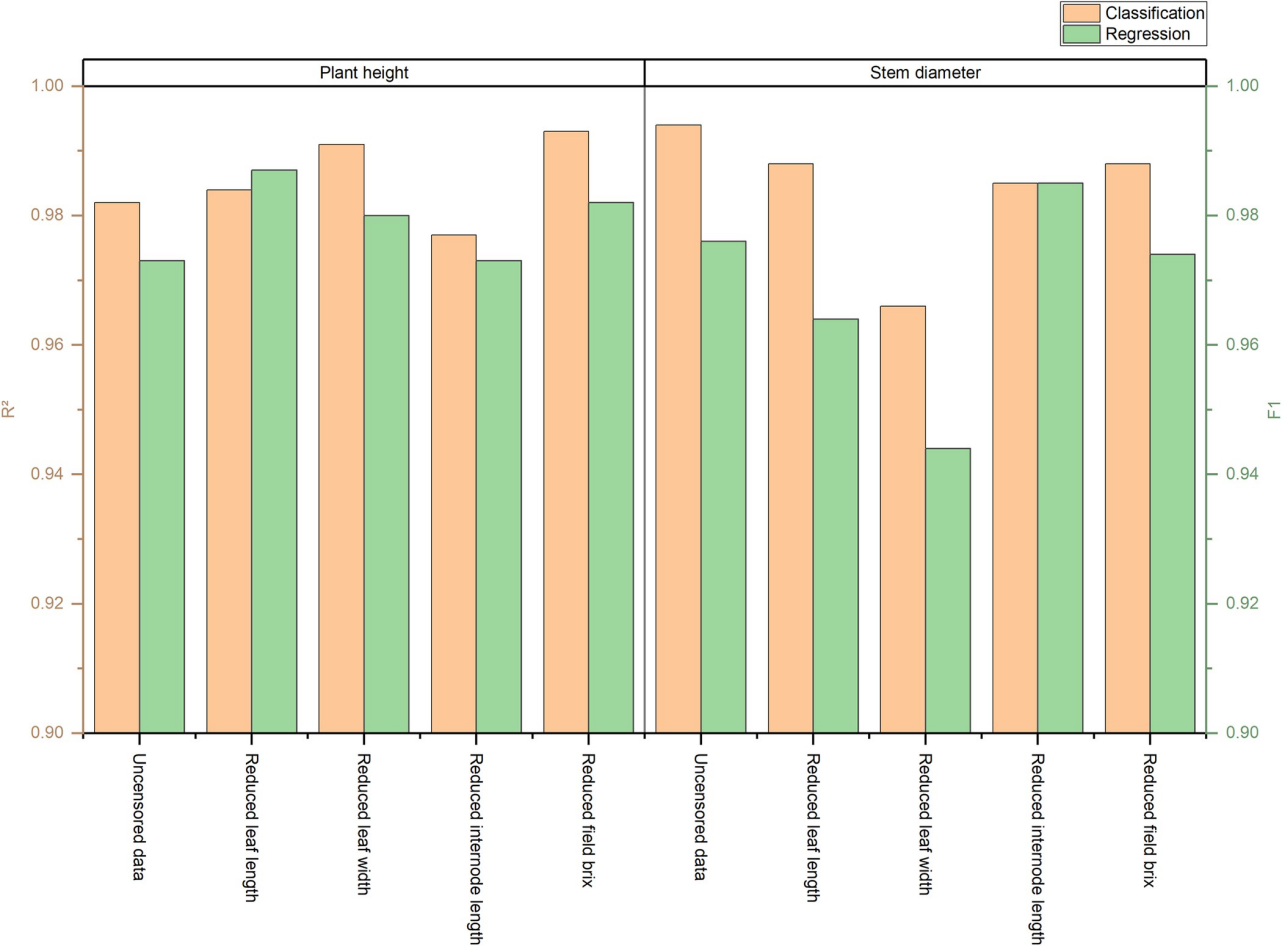
Performance of classification model and regression model after removing single influencing factors.

As depicted in Fig 7, upon the elimination of one of the influential variables, the classification models adopted the F1 value as a metric for evaluation, while the regression models employed the R^2^ value. Notably, the plant height classification model exhibited the most significant improvement in accuracy following the exclusion of the "leaf length" factor, attaining an F1 value of 0.987, thereby establishing itself as the definitive model for plant height classification in this study. Similarly, the stem diameter classification prediction model achieved its peak accuracy after diminishing the "internode length" factor, recording an F1 value of 0.985, and was consequently designated as the final model for stem diameter classification. Analogously, the plant height regression model observed a reduction in the "leaf length" factor’s R^2^ value to 0.991, thus serving as the culmination of the plant height regression prediction model. In the stem diameter regression model, the precision was enhanced to 0.988 following the reduction of either the "leaf length" or "field brix" factors, which ultimately constituted the final model. Consequently, the prediction models for both sugarcane plant height and stem diameter were conclusively determined.

## Discussion

### Comparison of model performance of different data processing methods

The experimental results reveal that in the classification and prediction experiments pertaining to sugarcane phenotypic traits (stem diameter and plant height), the accuracy of the machine learning-based intelligent prediction models utilizing raw data is comparatively low. While random sampling offers a marginal improvement in prediction accuracy, the utilization of the SMOTE-ENN combination sampling technique significantly enhances the accuracy of the prediction models to the highest level. Furthermore, our study observed that the inclusion of self-fitting data experiments led to a decrease in the accuracy of all models, suggesting that the integration of self-fitting data has a detrimental impact on the predictive accuracy of the models. However, despite this reduction in accuracy due to the addition of self-fitting data, the application of SMOTE-ENN combined sampling technology still managed to increase the predictive accuracy of the models. This underscores the effectiveness of the SMOTE-ENN combination sampling technique in significantly enhancing the predictive performance of the classification models.Therefore, by incorporating the SMOTE-ENN combined sampling technique to balance the dataset, we can not only improve the generalization ability of the models but also mitigate the challenges associated with data collection in the classification and prediction process for sugarcane stem diameter and plant height, ultimately enhancing the overall accuracy of the models.

### Performance comparison of different machine learning algorithms

The findings of our study indicate considerable variability in the performance of seven machine learning algorithms when employed in the construction of prediction models. Specifically, the models developed using the random forest and XGBoost algorithms outperformed those constructed with five other algorithms: decision tree, logistic regression, K-nearest neighbors (KNN), support vector machine (SVM), and backpropagation (BP) neural network. This superiority can be attributed to the random forest algorithm’s ability to mitigate overfitting risks and enhance prediction accuracy by integrating predictions from multiple decision trees, while XGBoost’s compatibility with diverse datasets and its parallel processing capabilities contribute to its strong performance. Notably, XGBoost exhibited a particularly robust ability in high-precision prediction, rendering it the preferred algorithm for the construction of prediction models for sugarcane phenotypic traits such as stem diameter and plant height. Among the remaining four algorithms, the decision tree algorithm demonstrated slightly lower performance compared to XGBoost and random forest, likely due to its proficiency in handling datasets with missing attributes and its faster execution during testing. The KNN-based prediction model achieved the highest accuracy, while the models constructed using logistic regression, SVM, and BP neural network algorithms exhibited accuracy levels below 0.6. While the KNN algorithm requires substantial computational resources, its simplicity in understanding and implementation is noteworthy. In contrast, the logistic regression algorithm struggles to capture the full range of data information, limiting its ability to handle complex data types, anomalies, and missing data. The SVM’s performance is sensitive to the choice of kernel function parameters and the presence of missing data, while the BP neural network faces challenges in determining the optimal number of hidden layers and nodes.

### Performance analysis of predictive models after adjusting influencing factors (stem diameter, plant height)

Following the elimination of the influential factor "leaf width," the decision tree, random forest, and XGBoost algorithms were employed to reconstruct the stem diameter classification prediction model. The comparison with the original model elucidated the pivotal role of "leaf width" in the prediction of sugarcane stem diameter classification. Our findings demonstrate that the accuracy of the reconstructed models improved by a range of 0.01 to 0.08 compared to the original model. Similarly, after excluding the influential factor "stem diameter," the decision tree and random forest algorithms were used to reconstruct the plant height prediction model. The comparison revealed a marginal accuracy difference between the new and original models, ranging from 0.01 to 0.02. Additionally, upon the elimination of "internode length," the XGBoost algorithm reconstructed the plant height prediction model, resulting in a negligible accuracy difference of only 0.01 compared to the original model.These research outcomes indicate that the inclusion or exclusion of individual influencing factors does not significantly alter the overall model accuracy. However, it is noteworthy that due to the relatively simplified experimental design focused on reducing influencing factors in this study, a comprehensive analysis of the impact of individual or multiple factors on model performance was not undertaken. Therefore, future research should delve deeper into this area.

### Analysis of the scalability of a sugarcane important phenotypic data prediction model based on multi-model and multi-task approach

To construct a practical and scalable predictive model for key phenotypic traits of sugarcane, this study employed the XGBoost algorithm, which is optimal for predicting phenotypic characteristics of sugarcane. We established field brix classification and regression models and conducted scalability experiments to enhance the model’s performance. The results of the scalability testing are presented in Table 9.

**Table 9:** Table of accuracy for field brix classification/regression prediction models.

Implicit variable	Data Manipulate	Classification				Regression			
**Field brix**		Accuracy	Recall	Precision	F1	MSE	RMSE	MAE	R^2^
	**Standardization**	0.359	0.359	0.352	0.352	0.031	0.177	0.137	0.062
	**Random oversampling**	0.829	0.829	0.827	0.823	0.009	0.095	0.041	0.826
	**SMOTE-ENN combination sampling**	0.957	0.957	0.956	0.956	0.092	0.303	0.084	0.969

In the field brix classification/regression prediction experiment, data were processed through methods such as data standardization, random oversampling, and combined sampling. Subsequently, XGBoost was employed to construct a sugarcane field brix classification/regression prediction model. Experimental results indicated that the performance of the field brix classification model was superior after combined sampling, achieving accuracy, recall, precision, and F1 scores of 0.957, 0.957, 0.956, and 0.956, respectively. Similarly, the field brix regression model demonstrated satisfactory predictive performance, with MSE, RMSE, MAE, and R2 values reaching 0.092, 0.302, 0.084, and 0.969, respectively, achieving precise prediction of field brix values. This demonstrates the scalability of the sugarcane important phenotypic data prediction model based on multi-model and multi-task, which is suitable for predicting different phenotypic characteristics of sugarcane.

## Conclusion

In addressing the challenges posed by the strong subjectivity and low prediction accuracy of classical empirical models, this study pioneers a comprehensive approach by developing a suite of significant sugarcane phenotype feature prediction models that harness the power of multiple integrated intelligent algorithms. Specifically, we leverage eight machine learning algorithms to formulate a novel method for indirectly forecasting yield based on sugarcane phenotype feature values. The methodology commences with data collection, where outliers are identified and mitigated, followed by data standardization. Subsequently, we employ random oversampling techniques and combined sampling methods to curate a robust data resource library, which serves as the foundation for model construction. Utilizing 555 sets of standardized and randomly sampled data as our training and testing sets, we deploy both non-ensemble and ensemble learning algorithms to construct an intelligent prediction model ensemble dedicated to wild sugarcane phenotypic traits. Furthermore, to enrich our dataset, we introduce a self-fitting data integration rule and employ the SMOTE-ENN method for combined sampling. By modeling in a consistent manner, we derive prediction results that are then compared against diverse modeling approaches. The analysis leads to the following key conclusions:

(1) Significant improvements in model performance were observed through a rigorous data preprocessing workflow encompassing data standardization, random oversampling, and SMOTE-ENN processing. Notably, in the stem diameter prediction model, following SMOTE-ENN processing, the already promising prediction results were further enhanced. Specifically, metrics such as accuracy, recall, precision, and F1 value exhibited substantial gains. The most notable enhancement was achieved by the KNN-based stem diameter classification prediction model, achieving improvements of 17.1%, 17.1%, 18.89%, and 17.65% respectively. Similarly, in plant height prediction, the KNN-based plant height classification prediction model yielded the highest performance gains, improving by 30.05%, 30.05%, 33.47%, and 32.44% in accuracy, recall, precision, and F1 value.

(2) In a comparative analysis with mainstream machine learning algorithms, including logistic regression, linear regression, KNN, support vector machine, BP neural network, decision tree, and random forest, the XGBoost algorithm emerged as the most effective for predicting stem diameter and plant height. When constructing the stem diameter prediction model, XGBoost significantly elevated accuracy, recall, precision, and F1 value to 0.976, 0.976, 0.977, and 0.976 respectively. This represented increases of 0.527, 0.527, 0.64, and 0.594 over the base models. Similarly, in the plant height prediction model, XGBoost optimized accuracy, recall, precision, and F1 value to 0.974, 0.974, 0.974, and 0.973 respectively, with gains of 0.555, 0.555, 0.55, and 0.576 in each metric.

(3) Upon augmenting the amount of self-generated data, the model’s performance exhibited minimal variations. As evident from the experimental results, the difference in model evaluation index values, both before and after the inclusion of self-fitting data, hovered around 0.005. This suggests that in the context of predicting crucial phenotypic data in sugarcane, the influence of incorporating self-fitting data on the model’s evaluation index value is negligible.

(4) To validate the model’s performance, we conducted a screening process of phenotypic features. By eliminating the "leaf length" factor in the plant height classification model, the F1 value ascended to 0.987. Similarly, the F1 value reached 0.985 after reducing the factor of internode length in the stem diameter classification prediction model. In the plant height regression model, the R^2^ value improved to 0.991 following the removal of the "leaf length" factor. Furthermore, in the stem diameter regression model, the accuracy enhanced to 0.988 after diminishing either "leaf length" or "field brix." Each model’s performance was further optimized based on the original framework, thus facilitating the final determination of influential phenotypic factors in sugarcane.

(5) This research is grounded in wild sugarcane phenotype data, leveraging sugarcane phenotypic characteristics as predictive factors to develop models for forecasting sugarcane stem diameter and plant height. Specifically, the models aim to predict the characteristic values of stem diameter and plant height post-planting for this sugarcane variety, thereby indirectly estimating sugarcane yield. Given the accuracy of these models in predicting post-planting stem diameter and height for specific traits, the application of the stem diameter prediction model in the hybridization of sugarcane varieties with taller plants and narrower stems holds the potential to enhance the likelihood of cultivating new sugarcane varieties with larger stem diameters and heights. Similarly, the plant height prediction model can be applied to the hybridization of sugarcane varieties with broader stems and shorter plants, increasing the probability of developing new sugarcane varieties with both larger stem diameters and plant heights. This approach offers a novel methodology for breeding new sugarcane varieties.
